# Iliac Vein Rupture During the Endovascular Treatment for May-Thurner Syndrome in a Previously Irradiated Pelvis

**DOI:** 10.7759/cureus.51318

**Published:** 2023-12-30

**Authors:** Lucas Leleu, Philippe Devaux, Carlo Caravaggio, Manuel Pirotte

**Affiliations:** 1 Thoracic and Vascular Surgery, Centre Hospitalier de Wallonie-Picarde, Tournai, BEL

**Keywords:** endovascular procedures, may-thurner syndrome, iliac vein rupture, iliac vein stenting, stent graft

## Abstract

May-Thurner syndrome (MTS) is a vascular condition for which endovascular management is commonly chosen. We report an unusual presentation of this syndrome in a patient with previous Wertheim hysterectomy and pelvic radiotherapy, characterized by bilateral leg swelling due to radiation-induced right iliac vein stenosis. Endovascular left iliac vein stenting was performed. During the procedure, an iliac vein rupture occurred after stenting and was successfully treated using a stent graft. Two months follow-up showed a significant reduction of the leg swelling and the patency of the iliac stents. This rare case highlights a potential major risk of iliac vein rupture during the endovascular procedure in an irradiated pelvis.

## Introduction

May-Thurner is a vascular condition characterized by the compression of the left common iliac vein (LCIV) under the right common iliac artery (RCIA) [1]. While clinical presentation commonly consists of unilateral left leg swelling, we report an unusual presentation with bilateral leg swelling in a patient with previous pelvic radiotherapy. A preoperative contrast-enhanced CT scan revealed an LCIV compression by the RCIA and a severe stenosis of the right common iliac vein, related to the previous radiotherapy. Endovascular treatment was proposed to the patient.

## Case presentation

We report the case of a 49-year-old woman with a history of cervical epidermoid carcinoma in 2017 treated by radical Wertheim hysterectomy and pelvic lymphadenectomy followed by pelvic radiotherapy. Two years after initial treatment she presented a recurrence on the right psoas successfully treated by radiochemotherapy. She also underwent splenectomy for splenic rupture following a colonoscopy.

**Figure 1:**
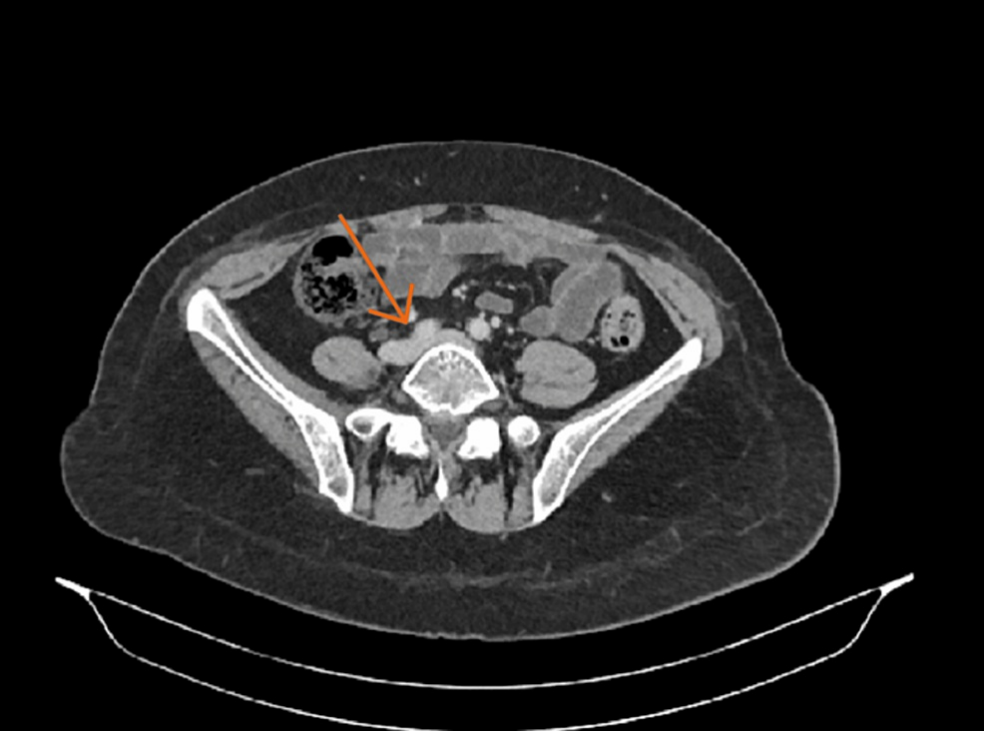
Preoperative CT scan showing a compression of the LCIV by the RCIA LCIV, left common iliac vein; RCIA, right common iliac artery

Three years after the last radiotherapy she presented with bilateral swelling of the lower limbs with a predominance on the left side. An investigation by peripheral venous Doppler ultrasonography revealed no deep vein thrombosis. A contrast-enhanced CT scan revealed a LCIV compression by the RCIA, also known as MTS, and a severe stenosis of the right common iliac vein (Figure 1). There was no evidence of malignant recurrence. Medical treatment by compression stockings was ineffective. Considering the patient history of previous pelvic procedures and radiotherapy, Iliac vein stenting was preferred over surgical treatment.

**Figure 2:**
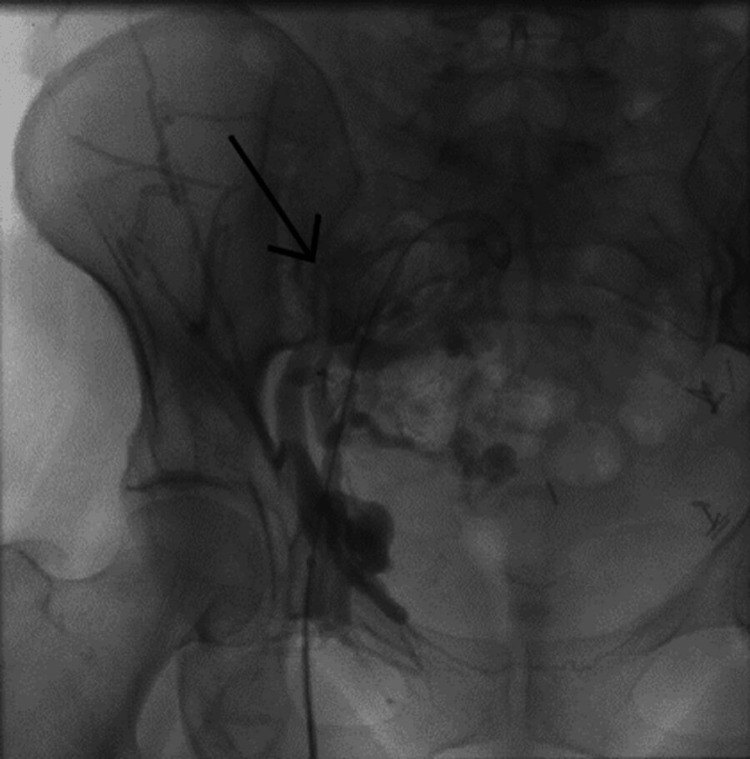
Venography showing an occluded right common iliac vein

**Figure 3:**
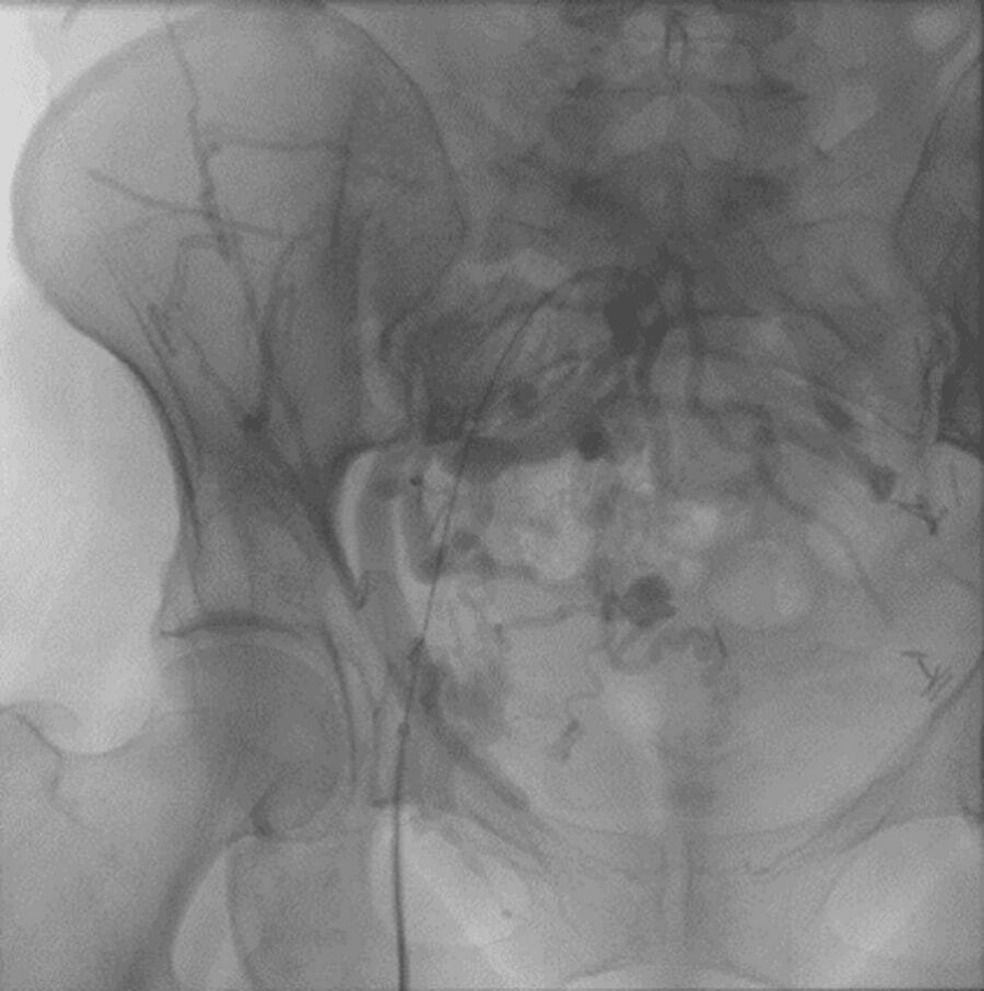
Pelvic collateral veins from right to left side

**Figure 4:**
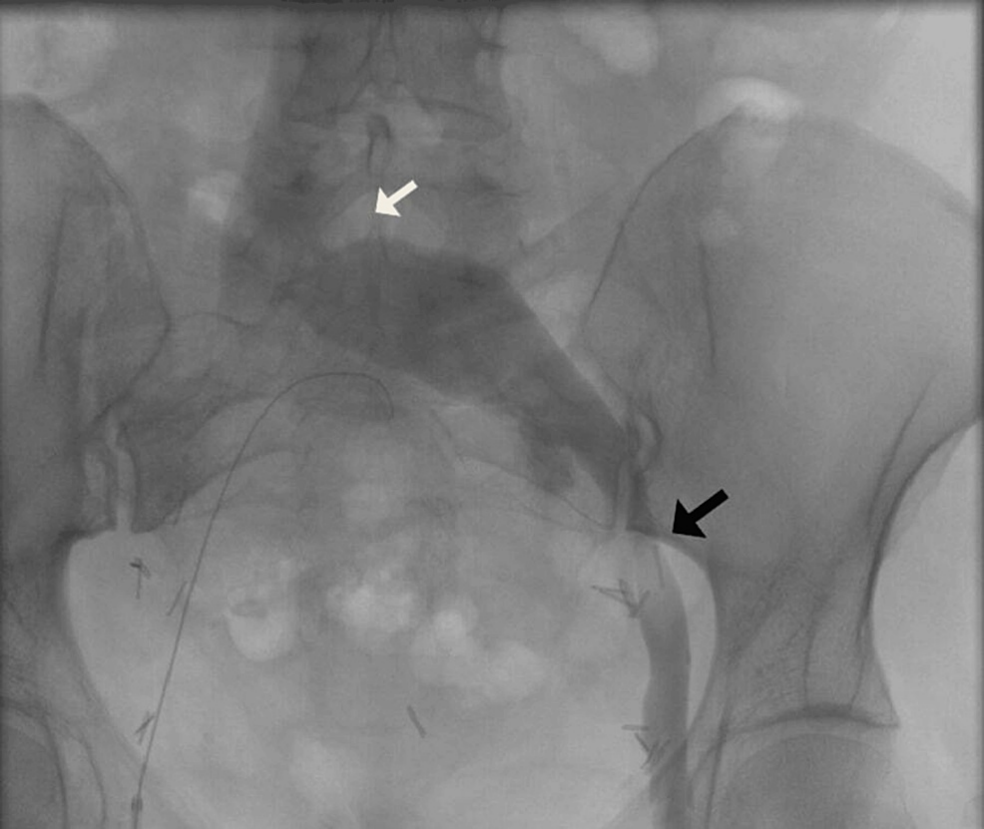
Compression of the LCIV by the RCIA (white arrow) and stenosis of the left external iliac vein (black arrow) LCIV, left common iliac vein; RCIA, right common iliac artery

The endovascular procedure was performed under spinal anesthesia. After heparinization with a 50 mg/kg bolus, we inserted a 5F sheath on the right common femoral vein. Venography showed an occluded right common iliac vein with pelvic collateralization to the left side (Figure 2 and Figure 3). Multiple attempts of catheterizations using a 0.035 hydrophilic-coated guide wire (Terumo) failed. A 10F sheath was inserted in the left common femoral vein and venography was performed, showing a clear compression of the LCIV and a stenosis on the left external iliac vein (Figure 4). Catheterization of the left iliac vein to the inferior vena cava was performed using a 0.035 hydrophilic-coated guide wire (roadrunner®, Cook).

Three stents were deployed starting with a 16/40 mm self-expanding nitinol stent (Venovo™) in the left external iliac vein. A second one (16/80 mm, OptiMed) was deployed to the common iliac vein with an overlap. Then a third one (18/80 mm Venovo™) to the inferior vena cava bifurcation.

**Figure 5:**
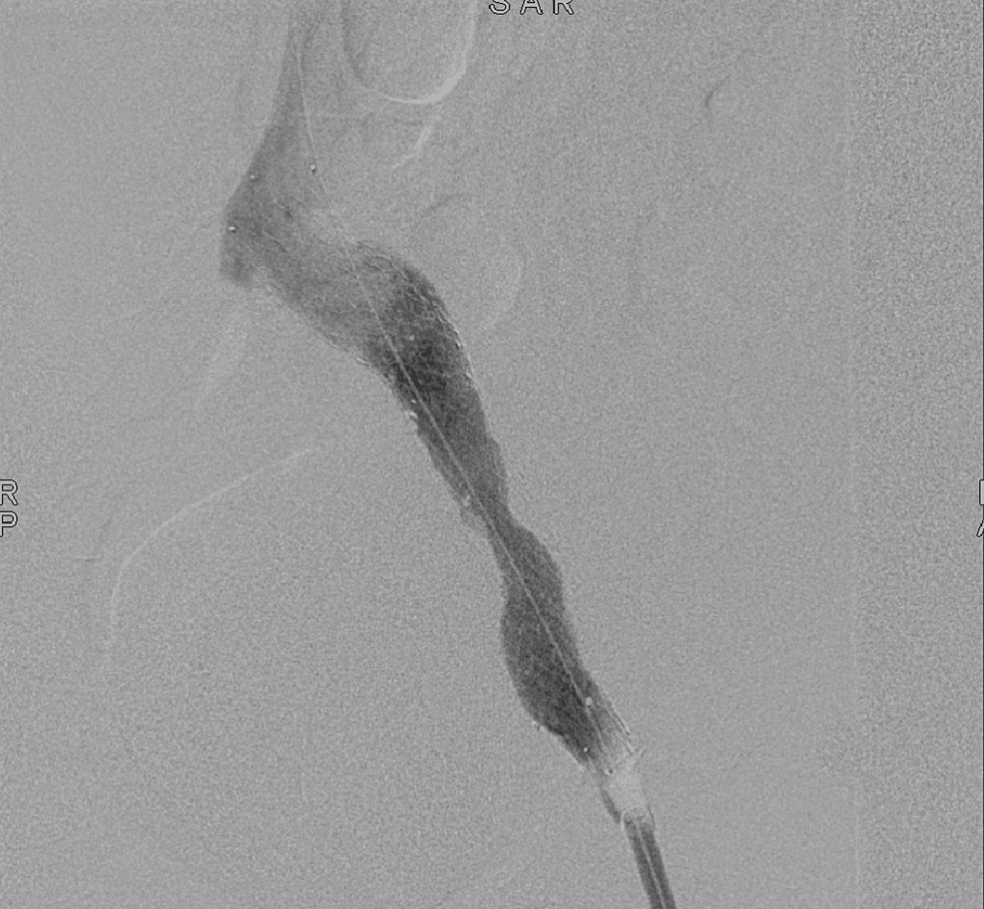
Venography performed after stenting showing an incomplete stent expansion

**Figure 6:**
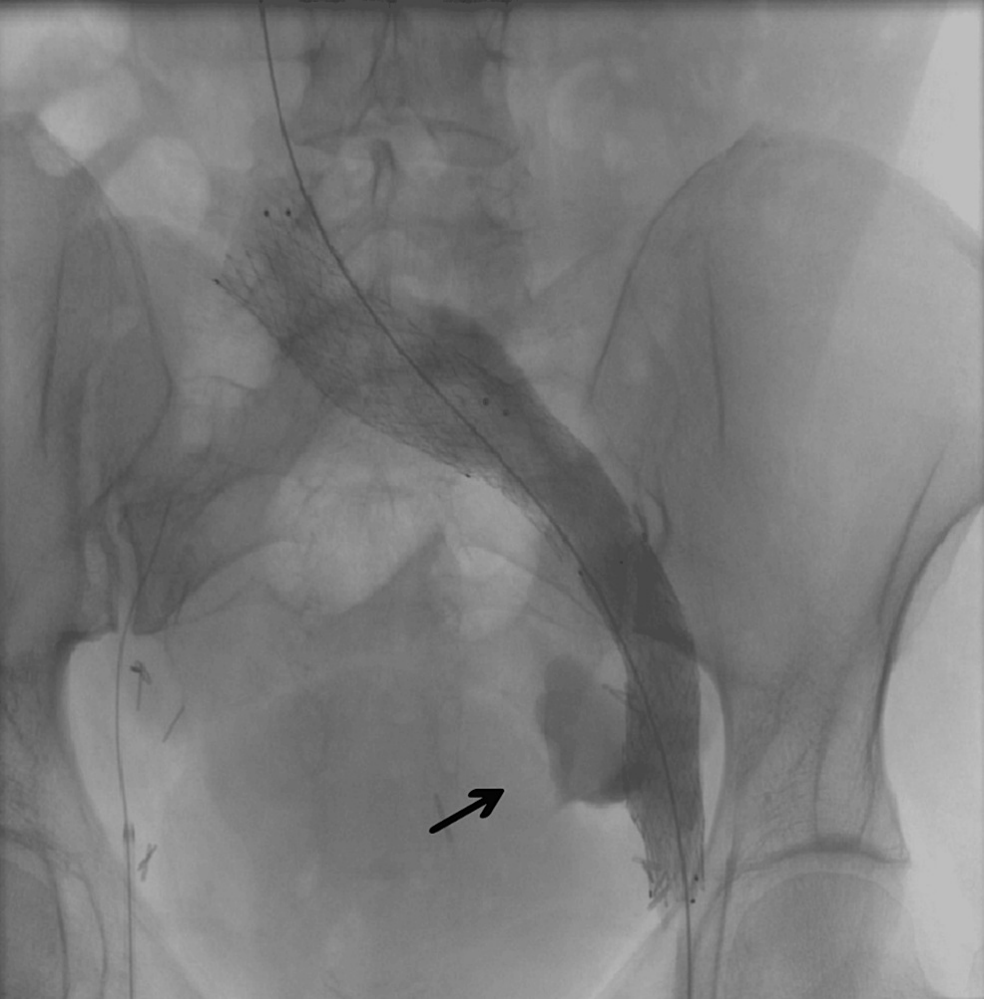
Venography performed post-stenting dilatation demonstrating extravasation

A 16 mm balloon was inflated because of an incomplete expansion on the middle part of the stenting (Figure 5). Venography showed contrast extravasation on the site of the previous dilatation. The 16 mm balloon was reinflated for a period of three minutes and then 10 minutes. Control venography showed a massive extravasation to the retroperitoneum and the balloon was immediately reinflated (Figure 6). The patient developed hemorrhagic shock. General anesthesia was performed; fluid resuscitation (crystalloids, blood transfusion) and vasopressor support were administrated to resuscitate the patient.

After stabilization, the balloon was deflated and the patient’s blood pressure immediately dropped, showing the extreme need to cover the vein rupture.

An 18/52 mm balloon-expandable covered stent (Bentley) was deployed. Nonetheless, there still was extravasation at the extremity of the stent, indirectly demonstrating the progression of the vein rupture. Due to the proximity of the hip joint and the risk of stent fracture, a new covered stent was not considered. Neither was direct surgical iliac vein repair for the previous major pelvic surgery and the two periods of pelvic radiotherapy, increasing the risk of further iatrogenic lesions. 

Aortic stent graft was considered to cover the whole site of rupture but none were available in our institution off the shelf. Two iliac extension limbs of aortic stent graft (Endurant® Medtronic) were delivered within three hours during which the balloon was still inflated at the site of extravasation.

**Figure 7:**
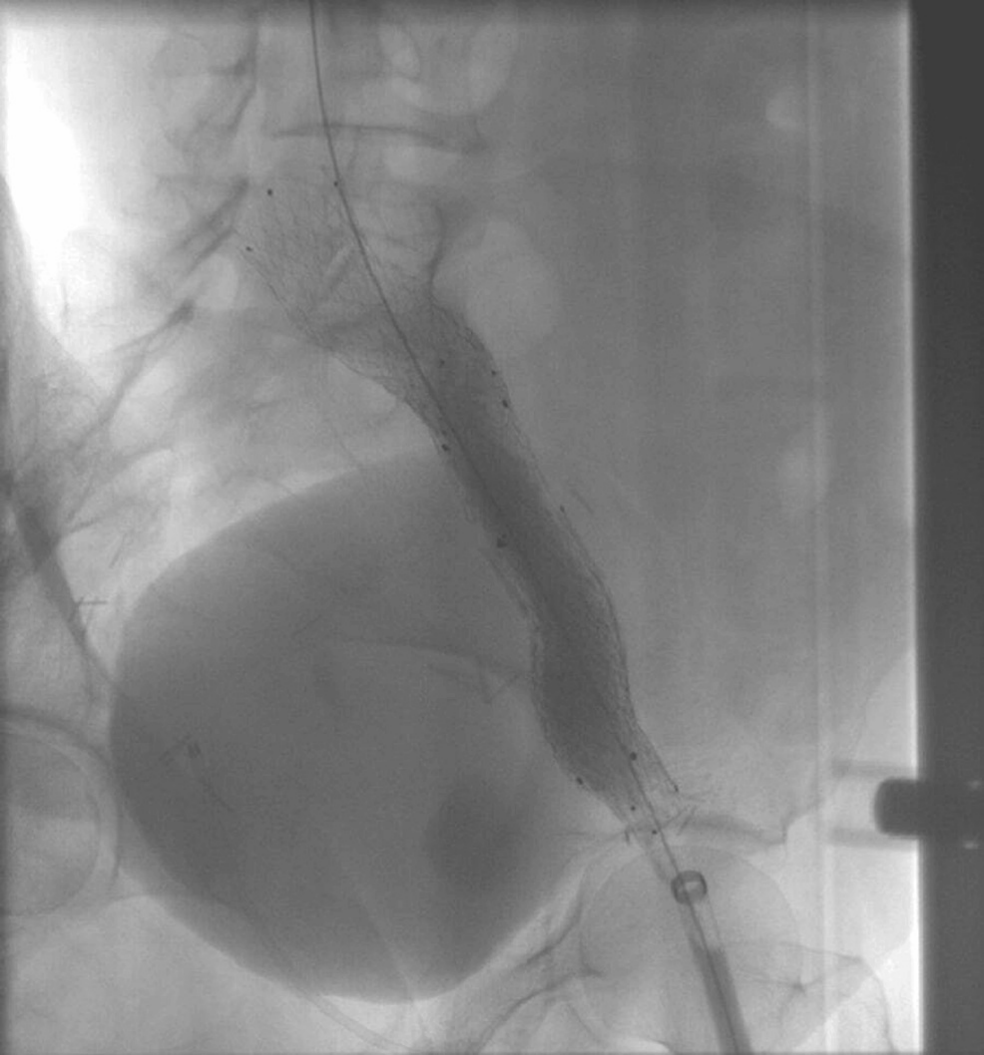
Final venography

The sheath was exchanged for an 18F, and the 20/82 mm stent graft was deployed. Further balloon hemostasis was performed on the distal part of the stent graft. The final venography showed the patency of the stenting and the absence of extravasation (Figure 7). The patient was transferred to the intensive care unit.

On day one following the procedure, the patient presented acute abdominal pain. A contrast-enhanced CT scan showed a voluminous hemoperitoneum, which required laparoscopic drainage, resulting in immediate postoperative pain relief. Therapeutic anticoagulation by LMWH (enoxaparine) was administered from day one until discharge.

The patient was discharged on day nine following the procedure. Discharge prescription consisted of oral anticoagulation (Sintrom) with a target INR of two to three and compression stockings.

Two-month postoperative follow-up showed a significant reduction of the lower limb swelling. A Doppler ultrasonography and contrast-enhanced CT scan confirmed the patency of the left iliofemoral veins.

## Discussion

MTS, or Cockett syndrome, is a vascular condition characterized by the compression of the left common iliac vein (LCIV) under the RCIA. Its clinical manifestation includes most commonly left lower limb swelling, venous claudication, or deep vein thrombosis and can be rarely associated with spontaneous iliac vein rupture [1]. Although MTS incidence is unclear, LCIV compression can be found in 37% to 66% of the asymptomatic population [2,3]. Clinical manifestation may occur when an additional disease renders this underlying compression symptomatic [2]. While venous Doppler ultrasonography is usually the first radiological modality, MTS is usually missed due to difficulties in observing the iliac vein segment. CT Venography has a high sensitivity and specificity for MTS diagnosis. However, the gold standard remains venography combined with intravascular ultrasound [4]. Endovascular treatment is preferred over surgical options considering the low morbidity and mortality rate for iliac vein stenting [1,4,5].

In this case, the patient had an unusual presentation of MTS considering the bilateral lower limbs swelling. This can be explained by the contralateral radiation-induced iliac vein occlusion resulting in pelvic collateralization to the left iliac veins. The concomitant existence of a majored venous outflow in the LICV and a "May-Thurner anatomy" led to this symptomatic MTS. Regarding the bilateral improvement of leg swelling, we believe that despite the catheterization failure on the right side, treating the left-side stenosis allowed the right to left venous collaterals to have enhanced drainage.

The growing number of patients benefitting from cancer therapy treatment, such as chemo and radiotherapy, has led to an increased consideration of cardiovascular side effects. Radiation-induced arterial disease is the result of vasa vasorum occlusions with necrosis and fibrosis of the medial tunic, fibrosis of the adventitial tunic, and accelerated atherosclerosis [6]. The mechanism of radiation-induced venous disease is yet to be described [7].

However, several cases of radiation-induced iliac vein stenosis have been described. Such stenosis was responsible for chronic venous obstruction resulting in deep vein thrombosis and leg swelling [8-13]. In every case, iliac vein stenting was performed, primarily or after thrombectomy or thrombolysis. Venous stenting for chronic iliofemoral venous obstruction is now the standard treatment in either non-thrombotic iliac vein lesions or post-thrombotic syndrome lesions [5]. Lee et al. showed that kissing stent for bilateral radiation-induced iliac vein stenosis is effective [13].

As recently mentioned by Xiang et al., iliac vein stenting in patients who underwent previous radiotherapy may differ from non-radiotherapy-related indications [12]. They reported an external iliac vein rupture after stent post-dilatation, which happened identically for our patient. While the optimal stent size for the iliac vein is unknown, Raju et al. suggested that the stent size should at least match the normal caliber of the vessel [14]. 14-16 mm stents are usually recommended for normal common iliac veins. Those two cases show that stent size should carefully be selected and post-dilatation limited. When radio-induced vein stenosis is suspected, intravascular ultrasonography (IVUS) should be used as it allows a more precise choice of stent size and landing segment while the reintervention rate is lower compared to the use of venography alone [15].

Iliac vein rupture, iatrogenic or traumatic, is a serious condition that could potentially be life-threatening. Management of such injuries includes open surgical repair and endovascular stenting. Several successful endovascular repairs for iliac vein rupture were reported, demonstrating its feasibility and effectiveness [12,16,17]. While uncommon, covered stent grafts have proven, in selected cases, to be effective and allow hemorrhage control in difficult situations [18].

## Conclusions

While unusual, MTS should be considered in bilateral lower limb swelling in patients with a history of pelvic irradiation. Endovascular stenting can be performed effectively and IVUS should be used to determine the stent size and landing zone. This case highlights a potentially major risk of iliac vein rupture during an endovascular procedure in an irradiated pelvis. Further research is needed for the understanding of radiation-induced vein disease.
